# Effects of Individual Discount Rate and Uncertainty Perception on Compliance with Containment Measures during the COVID-19 Pandemic

**DOI:** 10.3390/brainsci11101256

**Published:** 2021-09-22

**Authors:** Cinzia Calluso, Eleonora Grande, Alessia Erario, Annalisa Tosoni, Giorgia Committeri

**Affiliations:** 1 Department of Business and Management, LUISS Guido Carli University, Viale Romania 32, 00197 Roma, Italy; 2 Department of Neuroscience, Imaging and Clinical Sciences, Gabriele d’Annunzio University, Via dei Vestini 33, 66013 Chieti Scalo, Italy; eleonora.grande@studenti.unich.it (E.G.); alessia.erario@studenti.unich.it (A.E.); atosoni@unich.it (A.T.); giorgia.committeri@unich.it (G.C.)

**Keywords:** temporal discounting, intertemporal choice, risk perception, risky behavior, COVID-19 pandemic, containment measures, individual differences

## Abstract

Anti-contagion measures restricting individual freedom, such as social distancing and wearing a mask, are crucial to contain the COVID-19 pandemic. Decision-making patterns and attitudes about uncertainty can highly influence the adherence to these restrictive measures. Here we investigated the relationship between risky behavior and individual preferences for immediate vs. delayed reward, as indexed by temporal discounting (TD), as well as the association between these measures and confidence in the future, perceived risk and confidence in the containment measures. These measures were collected through an online survey administered on 353 participants at the end of the more restrictive phase of the first Italian lockdown. The results showed an unexpected inverse relationship between the individual pattern of choice preferences and risky behavior, with an overall greater adherence to containment measures in more discounter participants. These findings were interpreted in terms of a reframing process in which behaviors aimed at protecting oneself from contagion turn into immediate gains rather than losses. Interestingly, an excessive confidence in a better future was correlated with a higher tendency to assume risky behavior, thereby highlighting the downside of an overly and blindly optimistic view.

## 1. Introduction

The COVID-19 outbreak represents an unprecedented health emergency that has required and still requires adoption of strict containment measures in order to prevent an uncontrolled virus spread and the possible severe outcome associated with contagion. COVID-19 is severely affecting peoples’ lives on multiple levels and its consequences in terms of physical and psychological health are yet to be fully quantified. Nevertheless, some studies have begun to highlight the severity of the psychological effects determined by the pandemic outbreak. For example, moderate to severe psychological distress was reported in 53.8% of the study population involved in the recent study by Wang and colleagues on the consequences of the COVID-19 pandemic, and severe symptoms of anxiety were additionally reported in about one third of the participants [1]. Similarly, other studies reported high predominance of symptoms of depression, anxiety and stress [2] along with poor sleep quality and even PTSD symptoms [3] as a consequence of the COVID-19 pandemic. Additionally, the pandemic outbreak has produced—and is still producing—drastic effects on both the health system, which is facing a serious over-burdening, and the economic system, with a worldwide increase of poverty, social inequality and unemployment. 

To face the pandemic crisis, countries have adopted a series of strict containment measures such as social distancing, isolation, hand hygiene and mandatory indications of wearing protective masks. If, on one hand, these measures have a strong role in limiting the spread of the viral infection, they impose a massive change in lifestyle which has the potential of lasting for months or even years to come [4].

For these reasons, it is of crucial importance to understand the psychological mechanisms that facilitate or prevent the adherence and the commitment to these measures. Here, we sought to investigate this question by focusing on a specific decision-making phenomenon, known as temporal discounting (TD), that has been massively studied across several fields of investigation for its pervasiveness in daily life decision scenarios and implication for economic [5], clinical [6] and social domains [7]. TD reflects the decay over time of the subjective value of rewards and it is usually studied using intertemporal choice tasks, in which participants are required to choose between a smaller but immediately available monetary reward vs. a lager but temporally delayed one [8]. Considerable variations have been shown in the extent to which human participants select/prefer an immediate vs. a delayed reward, with the specific pattern of choice preference varying on a continuum between farsighted (i.e., strong preference for delayed reward) and discounter behavior (i.e., strong preference for immediate reward). Importantly, temporal discounting has been extensively described as a trait-like variable, for the specific features of being (i) relatively stable over time, while exhibiting developmental and experiential changes; (ii) correlated across different decision domains and type of rewards; (iii) associated with activity in particular brain regions and certain genetic markers [9]. Indeed, because of its trait-like characteristic, the discounting pattern of preference has been associated with the spectrum of addictive disorders [10,11] and several suboptimal life behaviors [12,13].

On this basis, the individual pattern of intertemporal choice may be strongly implicated in the compliance with containment measures during the current pandemic outbreak. For example, discounting behavior may encourage the pursuit of immediate benefits (i.e., sense of freedom) at the expense of long-term goals (i.e., reduction of the spread of the virus). In contrast, farsighted behavior might favor the adherence to restrictive measures due to the temporary postponement of short-term benefits (e.g., going out, visiting family and friends, traveling and enjoying freedom, etc.) in favor of a future greater benefit (e.g., health, end of the pandemic etc.)

Within this framework, the main aim of the present research was to investigate the association between the pattern of adherence to containment measures for COVID-19 and individual discounting functions. In particular, we sought to examine the extent to which patterns of individual preference for immediate vs. delayed rewards during intertemporal choices (i.e., farsighted vs. discounter) were associated with the individual risky behavior (Contagion Risk Index) against the restrictive containment measures during the first wave of the COVID-19 pandemic.

At the same time, it is important to note that the COVID-19 pandemic strongly impacts everyday life, by influencing the perceived level of safety and uncertainty about both the present and the future. For example, the perceived level of risk may impact upon the willingness to adhere to containment measures. A recent study, for example, employed a task measuring the delay discounting of containment measures as a function of the perceived risk, showing that the perception of risk influences the discounting of compliance with isolation [14]. Similarly, Cannito and colleagues reported an increase in the discounting of disposable masks as compared to money, suggesting that the feeling of scarcity experienced during the pandemic determined myopia (i.e., shortsighted decisions) when choosing a pandemic-relevant commodity (i.e., face masks) [15]. Based on this reasoning, as a second aim of the present study, we sought to examine the association between the individual risky behavior and the attitudes about uncertainty associated with the pandemic outbreak, such as confidence in the future, perceived risk and confidence in the containment measures. Finally, we also investigated whether these variables modulated the main relationship between the individual patterns of choice preferences (i.e., discounting rate) and the Contagion Risk Index.

For these purposes, we employed an online survey system to administer and collect data about preference patterns during intertemporal choices and self-reported measures of compliance with restrictive containment measures for COVID-19, as well as attitudes about uncertainty associated with the pandemic outbreak. The results suggest that both the pattern of intertemporal choices and the attitudes about uncertainty of the pandemic outbreak played a critical role in determining the level of adherence to the containment measures during the first Italian lockdown.

## 2. Materials and Methods

### 2.1. Participants 

The study involved a sample of 353 participants (251 females; due to an error in data acquisition procedure, no gender information was available for 47 of the study’s participants; age: 22.84 ± 6.02). In accordance with the ethical standards of the 1964 Declaration of Helsinki, all participants gave informed consent prior to study participation and were informed that data were stored and treated anonymously and of their right to discontinue participation at any time. The sample was prominently composed of master’s and bachelor’s degree students from the “G. D’Annunzio” University of Chieti-Pescara (employment level: students (81%), workers (11%), other (8%); Italy’s region of origin: Central (56%), South (40%), North (4%)). 

With respect to the educational level, most of the participants reported having high school degree (88%; 7% had a graduate degree, 3% postgraduate, 2% other). During the first lockdown, 80% of the participants reported being housed with their family of origin, 9% with roommates in shared flats, 7% with their partner and 4% alone. 

The great majority of participants (99%) reported not having contracted the COVID-19 infection, 1% suspected an infection. 2% of the sample reported to have completed a COVID-19 quarantine, as required by the Italian legislation in case of contact with infected people. A substantial change in their life routine was reported by 90% of participants.

### 2.2. Survey and Procedure

An online survey was administered at the end of the more restrictive phase of the Italian lockdown (May 2020) using the Qualtrics software (qualtrics.com accessed on 1 May 2020). After obtaining informed consent, a set of questions were administered to obtain demographical information, including age, gender, education level, working status and location. 

A second set of items were administered to collect information about the degree of adherence to containment measures and the level of risk assumed during the pandemic outbreak. These questions were specifically aimed at assessing the frequency of behaviors such as going out (e.g., grocery shopping, pharmacy, etc.), hand sanitation, use of protection devices (i.e., masks, gloves). 

A set of items were additionally administered to investigate the individual attitudes about uncertainty, including: the level of risk perception (i) before (prior to March 2020) and (ii) during the lockdown period (9 March–3 May, 2020); the level of confidence (iii) in the future and (iv) in the perceived effectiveness of the containment measures. A comprehensive list of the survey items is provided in Appendix A. Responses to the items were provided on a 10-point Likert scale. 

Anxiety and depression symptoms were also assessed using the State-Trait Anxiety Inventory (STAI; [16]) and the Center for Epidemiologic Studies Depression Scale (CES-D; [17]).

In the final section of the survey, a 27-item Monetary Choice questionnaire (MCQ-, [18,19]) was collected in which participants computed a series of hypothetical choices between two different amounts of money, one immediately available and one available at different time delays (e.g., “10 € now” or “25 in 7 days”). The MCQ was administered in its original form, with currency displayed in euros.

### 2.3. Analyses

#### 2.3.1. Contagion Risk Index and Attitudes about Pandemic-Related Uncertainty 

As described above, the questionnaire was divided into sub-sections, each assessing a specific aspect: risk perception during and before the first Italian lockdown period, confidence in the future, and confidence in the effectiveness of containment measures. In the section investigating attitudes about uncertainty, participants were asked to express their level of agreement with each item on a 10-point Likert scale (ranging from 1 for complete disagreement to 10 for complete agreement). Half of the items were formulated in a reverse manner and the relative score was converted before computation of the global score. For each participant, the global score of risk perception was computed by summing the individual scores of each item and then normalizing the resulting value in a range from 0 to 1. 

A similar analysis was adopted for the Risk Index. In this case, each item expressed the frequency of a certain behavior (i.e., going out for grocery shopping or other primary necessities, wearing a mask, wearing latex gloves, eluding lockdown restrictions, etc.) Since these scales were nonhomogeneous across different items, each item was first transformed into a scale ranging from 0 to 1 and then mediated across the items of the section to obtain a general index of contagion risk behavior (Contagion Risk Index).

#### 2.3.2. Discount Rate (k) Estimation

Choice data from the MCQ were analyzed using a standard routine based on R syntax [20]. The routine generates an individual’s rate of delay discounting (k) quantified using a hyperbolic discounting function described by Mazur (1987) [21], as follows:

(1) $$V=\frac{A}{\left(1+kD\right)}$$

where *V* is the present value of the delayed reward *A* at delay *D*, and *k* is a free parameter that determines the discount rate. High k values are associated with steep discounting functions, i.e., the preference for small immediate over larger delayed rewards or discounter behavior, while low k values are associated with higher delay tolerance, i.e., the tendency to prefer large delayed rewards or farsighted behavior (see [22,23,24,25,26,27]). A logarithmic transformation, prior to statistical testing, was performed to account for skewed distribution (Kolmogorov–Smirnov test of normality: *p* > 0.20). 

#### 2.3.3. Statistical Testing

The statistical association between the Contagion Risk Index and the individual discount rates (k), as well as the other collected measures, including the index of risk perception before and during the Italian lockdown, the index of confidence in the future and in containment measures, the scores of anxiety (STAI) and depression (CES-D), were assessed through Pearson correlation analyses and corrected for multiple comparisons using the False Discovery Rate (FDR) correction [28,29,30]. Additional correlations were computed between the discount rates (k) and all the other collected measures. Furthermore, in order to ensure that the results of the relationship between the discount rates (k) and the Contagion Risk Index were not guided by outliers, an additional robust regression [31,32] was conducted on these indices.

A series of moderation analyses were also conducted on the basis of the correlational results using the Process routine for SPSS [33,34]. Moderation analyses were performed using the Contagion Risk Index as dependent variable, the individual discount rates (k) as independent variable and the other variables, including the indices of attitudes about pandemic-related uncertainty (e.g., risk perception and confidence in the future) as moderators. All variables were z-transformed prior to statistical testing.

## 3. Results

As illustrated in Table 1, in which the mean value and standard deviation of all the measures collected in the survey are listed, a moderately low level of anxiety was recorded in our study sample (scores ≤ 40; [16]). In contrast with anxiety, analysis of the depression scale indicated that 49% of the study participants reported a score exceeding the clinical cut-off (scores ≥ 21; [17]), which highlights the presence of clinically relevant depression symptoms in almost half of the study population. 

### 3.1. Correlation Results

The results of correlation analyses conducted between the Contagion Risk Index and the other measures of interest are summarized in Table 2. The correlations between the discount rate (k) and the other variables are summarized in Table 3.

As quantified by the correlational results, with the only exception of the anxiety score, a statistically significant correlation was found between Contagion Risk Index and the other measures collected in the survey, including the pattern of preferences during the intertemporal choice task and the indices of attitudes about uncertainty of the pandemic situation. 

**Figure 1:**
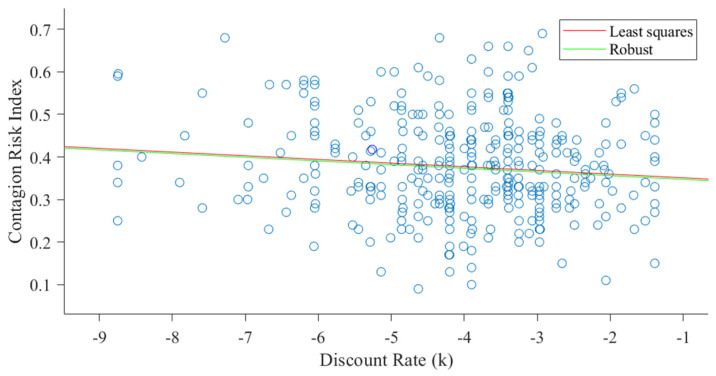
Results of the robust regression conducted using the Risk Index as dependent variable, the discount rate (k) as independent variable.

Regarding the decision-related pattern during the intertemporal choice task, we surprisingly found that discounting behavior was negatively associated with the Contagion Risk Index. This result was also confirmed by a robust regression (β = −0.09, *p* = 0.044), which was employed to ensure that the effect was not dragged by the presence of outliers (see Figure 1). This negative correlation indicated an increased tendency to assume contagion risks in participants with lower discounting rate, that is, participants with an overall tendency to prefer delayed larger vs. immediate smaller rewards. Therefore, contrary to our expectations, the results indicated that farsighted participants were more inclined to assume contagion risks with respect to discounter participants.

Similarly, the index of risk perception before (r = −0.24, *p* =0.004) and during (r = −0.16, *p* = 0.005) the lockdown was found to be negatively correlated with the Contagion Risk Index, thus suggesting that a lower perception of risk was associated with higher frequency of risky behavior. Along the same lines, proneness to risk was found to be negatively associated with the perceived effectiveness of the containment measures. In other words, participants with high confidence in the effectiveness of the containment measures were generally more inclined to adhere to the imposed restrictions and therefore less frequently reported risky behavioral pattern. Interestingly, the measure of confidence in a better future was positively correlated with the Contagion Risk Index, suggesting that participants with a positive feeling about the pandemic crisis and with the belief that the pandemic outbreak would soon come to an end appeared to assume a higher contagion risk. 

Finally, a negative correlation (r = −0.13, *p* = 0.02) was found between the Contagion Risk Index and the depression score (CES-D), indicating that participants with more severe symptoms of depression were less inclined to expose themselves to potential risk of contagion.

### 3.2. Moderation Analyses

The degree of association among the different measures and their combined modulatory effect upon the individual pattern of contagion risk behavior was assessed through moderation analyses conducted using the Contagion Risk Index as a dependent variable, the individual discount rate (k) as independent variable, and the other measures of interest as moderators. The subject-specific discount rate was treated as an independent variable on the basis of its trait-like definition, while the variables indexing the attitudes about pandemic-related uncertainty, such as risk perception, were treated as potential modulators (i.e., state-like) of the association between trait-like discounting functions and the pattern of risky behavior during the pandemic outbreak. 

As illustrated in Table 4, the only moderation analysis yielding a statistically significant interaction effect was the one conducted using the discount rates (k) as independent variable and the pre-lockdown perception of risk (β = 0.12, t = 2.19, *p* = 0.03) as moderator. These findings indicate that the negative relationship between individual discount rates and the contagion risk index was moderated by individual perception of contagion risk.

**Figure 2:**
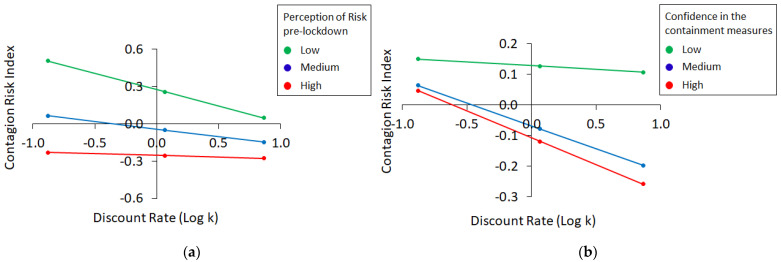
Results of the moderation models conducted using the Contagion Risk Index as dependent variable, the discount rate (k) as independent variable and the perception of risk pre-lockdown (**a**) or the confidence in the effectiveness of the containment measures (**b**) as moderators.

In particular, participants with a high level of risk perception (see Figure 2a, red line)—even before the beginning of the lockdown—manifested a tendency to assume a low level of risk, regardless of their individual discount rates (t = −0.29, *p* = 0.70). Said differently, independently from the pattern of preferences towards small/immediate vs. large/delayed rewards, participants that expressed a strong feeling of risk and concern for the pandemic condition were more inclined to adopt protection behavioral strategies and to adhere to containment measures. On the other hand, participants with a medium (Figure 2a, blue line; t = −2.36, *p* = 0.02) or low (Figure 2a, green line; t = −3.20, *p* = 0.002) level of risk perception were less inclined to adopt a protective behavioral pattern and this was more evident in participants with a low (farsighted) vs. high (discounters) discounting rates. To summarize, participants with an overall farsighted choice preference behavior and with a low or medium concern for the pandemic condition were associated with an increased tendency to assume contagion risks. 

A tendency toward statistical significance was also observed for the interaction effect obtained in the analysis conducted using the confidence in the effectiveness of containment measures (β = −0.09, t = −1.70, *p* = 0.09) as moderator. Despite the marginal statistical significance, this finding is particularly important for highlighting the potential role played by the level of confidence in the containment measures on the commitment and adherence to the same measures. More specifically, this marginally significant result indicates that participants with low confidence in the containment measures (Figure 2b, green line; t = −0.33, *p* = 0.74) were more inclined to assume a higher level of contagion risk and to avoid restrictions regardless of their individual discounting preferences. Conversely, in case of medium (Figure 2b, blue line; t = −2.67, *p* = 0.01) and high (Figure 2b, red line; t = −2.78, *p* = 0.01) level of confidence in the effectiveness of the containment measures, the degree of assumed risk changed according to the discounting preferences and was higher for farsighted than for discounter participants. In the remaining models, the interaction was found to be nonsignificant. 

## 4. Discussion

The pandemic outbreak offers a unique opportunity to study the impact of trait preferences during intertemporal choices on compliance with COVID-19 containment measures, with important implications for safety and prevention of uncontrolled virus spread. In this respect, indeed, it is worth noticing how the current framework of the pandemic outbreak allows to measure trait-like discounting functions in a substantially different context with respect to previous studies and extant literature.

Here we investigated the impact of inter-individual variability in choice preference during intertemporal decisions and the attitudes about uncertainty on the adherence to containment measures during the first wave of the COVID-19 pandemic.

Notwithstanding, the generally high adherence to containment measures and the overall depressive mood observed in our sample (see, e.g., [35] for consistent results on mood disorder symptoms during the pandemic), our results showed that the behavioral repertoire adopted in response to the pandemic outbreak in terms of protection from contagion risk and adherence to the containment measures was influenced by both the individual pattern of choice preference during intertemporal decisions and the attitudes about uncertainty.

In particular, here we found a significant negative correlation between the Contagion Risk Index and the individual discount rate: participants displaying a farsighted pattern of intertemporal preferences appeared to more frequently assume risky behaviors as compared to discounter participants, who were more compliant with restrictive measures and protective behavioral pattern. As mentioned above, the current result was quite surprising considering that steeper discounting functions are typically associated with the pursuit of immediate gratification within suboptimal lifestyle routines (e.g., savings behaviors [36], employment decisions [37], educational investment [38], energy conservation [7], and financial decisions [5]), sub-optimal life behaviors (e.g., obesity [39], smoking [40]) and clinical conditions (e.g., drug [10] and alcohol [11] abuse, gambling disorder [26]). In particular, we expected that participants with a preference bias for immediate but smaller rewards (i.e., discounters) would be more likely to engage in behavioral patterns associated with the pursuit of immediate gratification (e.g., going out and meeting people) at the expenses of greater future gratification [41]. Interestingly, Wismans and colleagues also found that discounting preferences were positively correlated with both hygiene and social distancing compliance, while a negative relationship was found in the case of impulsivity [42]. Hence, these results appear in line with our study in suggesting higher compliance with COVID-19 regulations associated with steeper discounting functions (i.e., discounter behavior).

However, while these results appear unexpected, it is important to note that as compared to classical paradigms for intertemporal choices, the present study context is of a different kind: when people adopt a farsighted course of action (i.e., compliance with containment measures) they are renouncing some degree of freedom for the sake of a potentially larger but also uncertain reward. Indeed, while the adoption of protective devices and social distancing reduce the probability of contracting the virus, they still do not guarantee the future reward of being safe. Conversely, when people adopt a pattern of non-/reduced adherence to the containment measures, they are securing an immediate reward of enjoying some degree of freedom at the expenses of a future reward that may not come regardless. 

On this basis, we propose that the pandemic outbreak might have acted as a reframing factor of mental representations of time and rewarding value of choice. In particular, one possible explanation and working hypothesis is that contagion-protective patterns have assumed a new rewarding significance. According to this hypothesis, the pandemic would have triggered a reframing process in which behaviors aimed at protecting oneself from contagion in the here and now represent a gain in terms of protection, rather than a loss in terms of freedom. In other words, the pandemic conditions might have induced a reframing of the choice context such that protective behaviors were now reinforced and rewarded rather than discarded and eluded.

Following the same argument, and in addition to the discounting pattern, we also found a significant association between the Contagion Risk Index and the mental representation of the pandemic outbreak. In particular, risk perception before and during the lockdown was negatively correlated with the risk index, indicating higher adherence to containment measures in participants with higher perception of the risks posed by COVID-19. 

Relevantly, the perception of risk before the lockdown was also found to modulate the association described above between the risk index and the pattern of intertemporal choices, so that participants with low or medium risk perception displayed a pattern of risky behaviors that depended on their individual discount rates (reduced risky behavior in discounters), while participants with high risk perception were more inclined to adhere to the containment measures, regardless of the intertemporal choice preference. This suggests that both discounting preferences and risk perception may play a role in limiting risky behavior by increasing the rewarding values of protective measures.

Albeit marginally significant, another factor that might influence the relationship between the Contagion Risk Index and the individual discounting preferences is the perceived reliability of the containment measures. While participants with lower confidence in containment measures tend to adopt risky behaviors regardless of their discounting preferences, high/medium confidence is positively associated with compliance with containment measures. Again, this result highlights the importance of the rewarding value of protecting behaviors: when containment measures are conceived as non-effective and useless, they probably lose their rewarding value/significance in terms of virus protection. 

Finally, we also observed that the risk index was positively correlated with confidence in a better future and the belief that the pandemic would soon come to an end. In particular, the results of a correlation analysis indicated that participants with a more optimistic idea of the future were more inclined to risk exposure. Therefore, despite the positive value on individual well-being of an optimistic view, these results underscore the possible detrimental effects of excessively optimistic views of the pandemic outbreak.

This study has some limitations that need to be acknowledged. First, the study was conducted on a very specific population of university students and this may pose some limitations in terms of generalization of the results. Secondly, while recent research has found results in line with those reported here [42], other studies have found the opposite pattern, namely that steeper delay discounting predicted poorer adherence to social distancing measures [43,44]. Therefore, further studies are necessary to better understand the relationship between adherence to containment measures and preferences in discounting behavior.

## 5. Conclusions

We can conclude that the individual pattern of choice preferences, as well as attitudes about the uncertainty related to the pandemic outbreak, such as future perception, perceived risk and confidence in the containment measures, are associated with the compliance with the protective measures and the pattern of contagion risk behavior. People who underestimate the risks, who believe that keeping distance, wearing masks and being cautious are pointless restrictions of their freedom, or who just believe that everything is going to be fine and the pandemic is only a flash in the pan, clearly take a greater number of risks, which has an impact upon our collective ability to protect ourselves from the virus. 

## Figures and Tables

**Table 1:** Mean values and standard deviations of the relevant measures collected in the survey.

	Mean Value	Standard Deviation
Depression (CES-D)	22.55	10.92
Anxiety (STAI)	26.59	3.08
Perception of Risk pre-lockdown	0.36	0.09
Perception of Risk during the lockdown	0.40	0.08
Confidence in the effectiveness of containment measures	0.43	0.09
Confidence in the Future	0.26	0.08
Discount Rate (k)	−4.00	1.46
Contagion Risk Index	0.38	0.11

**Table 2:** Overview of the correlation analyses conducted between the Contagion Risk Index and the other measures of interest.

	r	*p*-Value	FDR-adj *p*-Value
Depression (CES-D)	−0.133	0.012	0.017
Anxiety (STAI)	0.069	0.194	0.194
Perception of Risk pre-lockdown	−0.237	0.001	0.004
Perception of Risk during the lockdown	−0.163	0.002	0.005
Confidence in the effectiveness of containment measures	−0.146	0.006	0.011
Confidence in the Future	0.174	0.001	0.004
Discount Rate (k)	−0.110	0.039	0.046

**Table 3:** Overview of the correlation analyses conducted between the discount rates (k) and the other measures of interest.

	r	*p*-Value	FDR-adj *p*-Value
Depression (CES-D)	0.08	0.16	0.32
Anxiety (STAI)	−0.13	0.02	0.14
Perception of Risk pre-lockdown	−0.05	0.35	0.41
Perception of Risk during the lockdown	−0.07	0.18	0.32
Confidence in the effectiveness of containment measures	−0.04	0.47	0.47
Confidence in the Future	−0.05	0.32	0.41

**Table 4:** Overview of the results of the moderation analyses.

Perception of Risk Pre-Lockdown			
	β	SE	t
Discount Rate (k)	−0.14	0.05	−2.73 **
Risk Perception pre-lockdown	−0.26	0.05	−4.97 ***
Interaction	0.12	0.05	2.19 *
**Perception of Risk during the Lockdown**			
	β	se	t
Discount Rate (k)	−0.13	0.05	−2.40 *
Risk Perception during the lockdown	−0.17	0.05	−3.30 ***
Interaction	0.03	0.06	0.60
**Confidence in the Effectiveness of Containment Measures**			
	β	se	t
Discount Rate (k)	−0.11	0.05	−2.07 *
Confidence Containment Measures	−0.14	0.05	−2.66 **
Interaction	−0.09	0.05	−1.70 ^†^
**Confidence in the Future**			
	β	se	t
Discount Rate (k)	−0.10	0.05	−1.90 ^†^
Confidence in the Future	0.17	0.05	3.19 ***
Interaction	−0.06	0.05	−1.11
**Depression (CES-D)**			
	β	se	t
Discount Rate (k)	−0.10	0.05	−1.90 ^†^
CESD	−0.13	0.05	−2.38 *
Interaction	−0.01	0.05	−0.10
**Anxiety (STAI)**			
	β	se	t
Discount Rate (k)	−0.11	0.05	−1.96 *
STAI	0.05	0.05	1.00
Interaction	0.02	0.06	0.40

*** *p* < 0.001; ** *p* < 0.01; * *p* < 0.05; ^†^  *p* < 0.1.

## Data Availability

The datasets used and/or analyzed during the current study are available at http://doi.org/10.17632/8k8yk2hy3k.1. accessed on 18 September 2021.

## Appendix A

**Table A1:** List of the items included in the questionnaire.

(Risk Perception Before the Lockdown) *
Prima del 9 Marzo—data di inizio del lockdown, su una scala da 1 a 10 quanto ritenevi che:
Le misure di contenimento dell’epidemia fossero utili/necessarie Le misure di contenimento dell’epidemia fossero eccessive Il COVID-19 fosse poco più che un’influenza La situazione fosse gestita con eccessivo allarmismo I governi stessero tardando nel dare inizio alle misure di contenimento dell’epidemia. La situazione sarebbe presto andata fuori controllo Che non stessimo facendo abbastanza per contenere i contagi Che il COVID-19 si sarebbe presto rivelato un fuoco di paglia
**(Risk Perception During the Lockdown) ***
**Dopo il 9 Marzo—data di inizio del lock-down, su una scala da 1 a 10 quanto ritenevi che:**
Le misure di contenimento dell’epidemia fossero utili/necessarie Le misure di contenimento dell’epidemia fossero eccessive Il COVID-19 fosse poco più che un’influenza La situazione fosse gestita con eccessivo allarmismo I governi stessero tardando nel dare inizio alle misure di contenimento dell’epidemia. La situazione sarebbe presto andata fuori controllo Che non stessimo facendo abbastanza per contenere i contagi Che il COVID-19 si sarebbe presto rivelato un fuoco di paglia
**(Confidence in the Future) ***
Quando pensi al futuro ed alla fine del lockdown, su una scala da 1 a 10 quanto ritieni che:
Andrà tutto bene Non riusciremo a sconfiggere il virus Presto torneremo alle nostre vite normali Questa situazione non si risolverà a breve Il futuro è pieno di speranza Il futuro è inaspettato ed imprevedibile Ci sono molte cose che vorrei fare alla fine del lockdown È inutile pensare alla fine del lock down, nulla sarà più come prima
**(Confidence in the Containment Measures) ***
Quando pensi al lockdown, su una scala da 1 a 10 quanto ritieni che:
Stiamo facendo tutto il possibile per contenere i contagi Tutte queste misure preventive sono inutili Preferisco avere pazienza e sopportare il lockdown per prevenire la possibilità di contagiarmi Il lockdown è un’inaccettabile privazione della mia libertà
**(Contagion Risk Index) ***
In relazione al periodo di lockdown, indica per ciascun comportamento quanto spesso ti è capitato di metterlo in atto
Indossare la mascherina (a) Ogni volta che esco di casa (b) La maggior parte delle volte in cui esco di casa (c) Solo quando entro in luoghi chiusi (es., supermercato, farmacia etc.) (d) Qualche volta (e) Mai Indossare i guanti (a) Ogni volta che esco di casa (b) La maggior parte delle volte in cui esco di casa (c) Solo quando entro in luoghi chiusi (es., supermercato, farmacia etc.) (d) Qualche volta (e) Mai Quanto spesso ti lavi/disinfetti le mani: (a) Raramente (b) La mattina/sera e prima dei pasti (c) La mattina/sera, prima dei pasti ed ogni volta che rientro a casa (d) Ogni volta che tocco qualcosa che penso possa essere contaminato (e) Molto frequentemente, anche senza aver toccato qualcosa di potenzialmente contaminato Quanto spesso sei uscito di casa per fare la spesa: (a) Una volta a settimana o meno (b) Circa 2–3 volte a settimana (c) Più di 4 volte a settimana (d) Tutti i giorni (e) Non sono io ad occuparmi della spesa (f) Ordino la spesa online Quanto spesso sei uscito di casa per occuparti di altre faccende di prima necessità (es., andare in farmacia, medico di base, etc.) che NON includono fare la spesa: (a) Una volta a settimana o meno (b) Circa 2–3 volte a settimana (c) Più di 4 volte a settimana (d) Tutti i giorni (e) Non sono io ad occuparmi di queste faccende (f) Quando possibile, uso metodi alternativi (es., contatto il medico per telefono, ordino i farmaci online etc.) Nel corso del periodo di lockdown, ti è mai capitato di infrangere la quarantena, ovvero di uscire di casa per ragioni non previste dal decreto in vigore (es., ragioni di prima necessità, ragioni lavorative, salute etc.)? (a) Mai (b) Una sola volta (c) Qualche volta (d) Spesso (e) Molto spesso

* The name of each measure was not visible.

## References

[1] Wang C., Pan R., Wan X., Tan Y., Xu L., Ho C.S., Ho R.C. (2020). Immediate Psychological Responses and Associated Factors during the Initial Stage of the 2019 Coronavirus Disease (COVID-19) Epidemic among the General Population in China. Int. J. Environ. Res. Public Health.

[2] Mazza C., Ricci E., Biondi S., Colasanti M., Ferracuti S., Napoli C., Roma P. (2020). A nationwide survey of psychological distress among italian people during the covid-19 pandemic: Immediate psychological responses and associated factors. Int. J. Environ. Res. Public Health.

[3] Casagrande M., Favieri F., Tambelli R., Forte G. (2020). The enemy who sealed the world: Effects quarantine due to the COVID-19 on sleep quality, anxiety, and psychological distress in the Italian population. Sleep Med..

[4] Coroiu A., Moran C., Campbell T., Geller A.C. (2020). Barriers and facilitators of adherence to social distancing recommendations during COVID- 19 among a large international sample of adults. PLoS ONE.

[5] Frydman C., Camerer C.F. (2016). The Psychology and Neuroscience of Financial Decision Making. Trends Cogn. Sci..

[6] Bickel W.K., Miller M.L., Yi R., Kowal B.P., Lindquist D.M., Pitcock J.A., Diana M., Pitcock J.A. (2007). Behavioral and Neuroeconomics of Drug Addiction: Competing Neural Systems and Temporal Discounting Processes. Drug Alcohol Depend..

[7] Allcott H., Greenstone M. (2012). Is There an Energy Efficiency Gap?. J. Econ. Perspect..

[8] Berns G.S., Laibson D., Loewenstein G. (2007). Intertemporal choice--toward an integrative framework. Trends Cogn. Sci..

[9] Odum A.L. (2011). Delay discounting: Trait variable?. Behav. Process..

[10] Kirby K.N., Petry N.M. (2004). Heroin and cocaine abusers have higher discount rates for delayed rewards than alcoholics or non-drug-using controls. Addiction.

[11] Petry N.M. (2001). Delay discounting of money and alcohol in actively using alcoholics, currently abstinent alcoholics, and controls. Psychopharmacology.

[12] Yi R., Matusiewicz A.K., Tyson A. (2016). Delay Discounting and Preference Reversals by Cigarette Smokers. Psychol. Rec..

[13] Weller R.E., Cook E.W., Avsar K.B., Cox J.E. (2008). Obese women show greater delay discounting than healthy-weight women. Appetite.

[14] Nese M., Riboli G., Brighetti G., Sassi V., Camela E., Caselli G., Sassaroli S., Borlimi R. (2020). Delay discounting of compliance with containment measures during the COVID-19 outbreak: A survey of the Italian population. J. Public Health.

[15] Cannito L., Anzani S., Bortolotti A., Palumbo R., Ceccato I., Di Crosta A., Di Domenico A., Palumbo R. (2021). Temporal discounting of money and face masks during the covid-19 pandemic: The role of hoarding level. Front. Psychol..

[16] Spielberger C.D., Gorsuch R.L., Lushene R.E. (1970). Manual for the State-Trait Anxiety Inventory.

[17] Radloff L.S. (1977). The CES-D Scale: A Self-Report Depression Scale for Research in the General Population. Appl. Psychol. Meas..

[18] Kirby K.N., Marakovic N.N. (1996). Delay-discounting probabilistic rewards: Rates decrease as amounts increase. Psychon. Bull. Rev..

[19] Kirby K.N., Petry N.M., Bickel W.K. (1999). Heroin addicts have higher discount rates for delayed rewards than non-drug-using controls. J. Exp. Psychol. Gen. Psychol..

[20] Gray J.C., Amlung M.T., Palmer A.A., MacKillop J. (2016). Syntax for calculation of discounting indices from the monetary choice questionnaire and probability discounting questionnaire. J. Exp. Anal. Behav..

[21] Mazur J.E., Commons M., Mazur J., Nevin J., Rachlin H. (1987). An adjusting procedure for studying delayed reinforcement. Quantitative Analysis of Behavior: Vol. 5. The Effect of Delay and of Intervening Events of Reinforcement Value.

[22] Calluso C., Committeri G., Pezzulo G., Lepora N., Tosoni A. (2015). Analysis of hand kinematics reveals inter-individual differences in intertemporal decision dynamics. Exp. Brain Res..

[23] Calluso C., Tosoni A., Pezzulo G., Spadone S., Committeri G. (2015). Interindividual variability in functional connectivity as long-term correlate of temporal discounting. PLoS ONE.

[24] Calluso C., Tosoni A., Fortunato G., Committeri G. (2017). Can you change my preferences? Effect of social influence on intertemporal choice behavior. Behav. Brain Res..

[25] Calluso C., Tosoni A., Cannito L., Committeri G. (2019). Concreteness and emotional valence of episodic future thinking (EFT) independently affect the dynamics of intertemporal decisions. PLoS ONE.

[26] Calluso C., Pettorruso M., Tosoni A., Carenti M.L., Cannito L., Martinotti G., Di Giannantonio M., Committeri G. (2020). Cognitive dynamics of intertemporal choice in gambling disorder. Addict. Behav..

[27] Calluso C., Zandi M.A., Devetag M.G. (2020). Cognitive Dynamics of Religiosity and Intertemporal Choice Behavior. J. Cross-Cult. Psychol..

[28] Benjamini Y., Hochberg Y. (1995). Controlling the False Discovery Rate: A Practical and Powerful Approach to Multiple Testing. J. R. Stat. Soc. Ser. B.

[29] Benjamini Y., Hochberg Y. (2000). On the Adaptive Control of the False Discovery Rate in Multiple Testing with Independent Statistics. J. Educ. Behav. Stat..

[30] Benjamini Y., Krieger A.M., Yekutieli D. (2006). Adaptive linear step-up procedures that control the false discovery rate. Biometrika.

[31] O’Leary D.P. (1990). Robust regression computation using iteratively reweighted least squares. Siam J. Matrix Anal. Appl..

[32] Holland P.W., Welsch R.E. (1977). Robust regression using iteratively reweighted least-squares. Commun. Stat.-Theory Methods.

[33] Hayes A.F. (2014). Comparing Conditional Effects in Moderated Multiple Regression: Implementation Using PROCESS for SPSS and SAS.

[34] (2019). IBM SPSS Statistics.

[35] Charles N.E., Strong S.J., Burns L.C., Bullerjahn M.R., Serafine K.M. (2021). Increased mood disorder symptoms, perceived stress, and alcohol use among college students during the COVID-19 pandemic. Psychiatry Res..

[36] Meier S., Sprenger C. (2010). Present-Biased Preferences and Credit Card Borrowing. Am. Econ. J. Appl. Econ..

[37] Schoenfelder T.E., Hantula D. (2003). A job with a future? Delay discounting, magnitude effects, and domain independence of utility for career decisions. J. Vocat. Behav..

[38] Castillo M., Ferraro P.J., Jordan J.L., Petrie R. (2011). The today and tomorrow of kids: Time preferences and educational outcomes of children. J. Public Econ..

[39] Davis C., Patte K., Curtis C., Reid C. (2010). Immediate pleasures and future consequences. A neuropsychological study of binge eating and obesity. Appetite.

[40] Mitchell S.H. (1999). Measures of impulsivity in cigarette smokers and non-smokers. Psychopharmacology.

[41] Brooks S.K., Webster R.K., Smith L.E., Woodland L., Wessely S., Greenberg N., Rubin G.J. (2020). The psychological impact of quarantine and how to reduce it: Rapid review of the evidence. Lancet.

[42] Wismans A., Letina S., Wennberg K., Thurik R., Baptista R., Burke A., Dejardin M., Janssen F., Santarelli E., Torrès O. (2021). The role of impulsivity and delay discounting in student compliance with COVID-19 protective measures. Pers. Individ. Dif..

[43] Lloyd A., McKay R., Hartman T., Vincent B.T., Murphy J., Gibson Miller J., Levita L., Bennett K.M., McBride O., Martinez A.P. (2021). Delay discounting and under-valuing of recent information predict poorer adherence to social distancing measures during the COVID-19 pandemic. Sci. Rep..

[44] DeAngelis B.N., Ben Salah A., al’Absi M. (2021). Stress and COVID-19 related behaviours: The mediating role of delay discounting. Stress Health.

